# Influence of Rotation Increments on Imaging Performance for a Rotatory Dual-Head PET System

**DOI:** 10.1155/2017/8615086

**Published:** 2017-01-05

**Authors:** Fanzhen Meng, Xu Cao, Xuezhou Cao, Jianxun Wang, Liang Li, Xueli Chen, Shouping Zhu, Jimin Liang

**Affiliations:** Engineering Research Center of Molecular and Neuro Imaging of Ministry of Education, School of Life Science and Technology, Xidian University, Xi'an, Shaanxi 710071, China

## Abstract

For a rotatory dual-head positron emission tomography (PET) system, how to determine the rotation increments is an open problem. In this study, we simulated the characteristics of a rotatory dual-head PET system. The influences of different rotation increments were compared and analyzed. Based on this simulation, the imaging performance of a prototype system was verified. A reconstruction flowchart was proposed based on a precalculated system response matrix (SRM). The SRM made the relationships between the voxels and lines of response (LORs) fixed; therefore, we added the interpolation method into the flowchart. Five metrics, including spatial resolution, normalized mean squared error (NMSE), peak signal-to-noise ratio (PSNR), contrast-to-noise (CNR), and structure similarity (SSIM), were applied to assess the reconstructed image quality. The results indicated that the 60° rotation increments with the bilinear interpolation had advantages in resolution, PSNR, NMSE, and SSIM. In terms of CNR, the 90° rotation increments were better than other increments. In addition, the reconstructed images of 90° rotation increments were also flatter than that of 60° increments. Therefore, both the 60° and 90° rotation increments could be used in the real experiments, and which one to choose may depend on the application requirement.

## 1. Introduction

Dual-head positron emission tomography (PET) systems have emerged in recent years at a lower cost and complexity [1–5]. For a stationary dual-head PET system [3, 6–8], the incomplete angle information degrades the spatial resolution perpendicular to the detector heads even if the iteration algorithms are used [9–12]. Some researchers have adopted the rotation operation to solve this problem [13–15]. There are mainly two schemes, rotating the detectors or rotating the object. For example, in 2006, Bruyndonckx et al. have proposed a prototype PET scanner in which the two detector heads are installed on a rotating platform [16]. In 2010, a small-animal PET system including a rotation platform in the middle of the detector heads has been addressed [2]. Nevertheless, how to determine the rotation increments is also an open problem. Efthimiou et al. have evaluated the performance of a rotatory dual-head system by a series of angles [17], and in their work the system response matrix (SRM) is geometrically estimated which is not accurate and is apt to cause depth of interaction (DOI) blurring near the field of view (FOV) boundaries [8, 13, 17].

The quality of the reconstructed images depends on the accurate modeling of relationships between the object space and measurement space [18]. The Monte Carlo (MC) simulation can accurately simulate the system model through a feasible empirical setup [19], but the SRM is precalculated and prestored in the computer. For the precalculated SRM, the relationships between the voxels (object space) and lines of response (LORs) (measurement space) are fixed. Once the system rotates an angle, the FOV should be rotated by the same angle to match with the precalculated SRM. When the rotation angle is not a multiple of 90°, an image interpolation operation is needed to build the relationships of the reconstructed images before and after rotation. In this study, we incorporate the interpolation methods [20] including the nearest neighbor interpolation, bicubic interpolation, and bilinear interpolation into the iteration reconstruction process. The different interpolation methods are compared to find the optimal interpolation, whose errors have minimum influence on the optimization cost function.

The purpose of this study is to analyze the influences of different rotation increments on the imaging performances of a rotatory dual-head PET system based on an accurate SRM. The rest of the article is organized as follows. In Section 2, we introduce the simulation of the dual-head PET system and describe the reconstruction method and experiment schemes. The results are shown in Section 3. Finally, the discussion and conclusion are in Section 4.

## 2. Materials and Methods

### 2.1. Simulation Scheme

#### 2.1.1. Simulation of Dual-Head PET System

**Figure 1:**
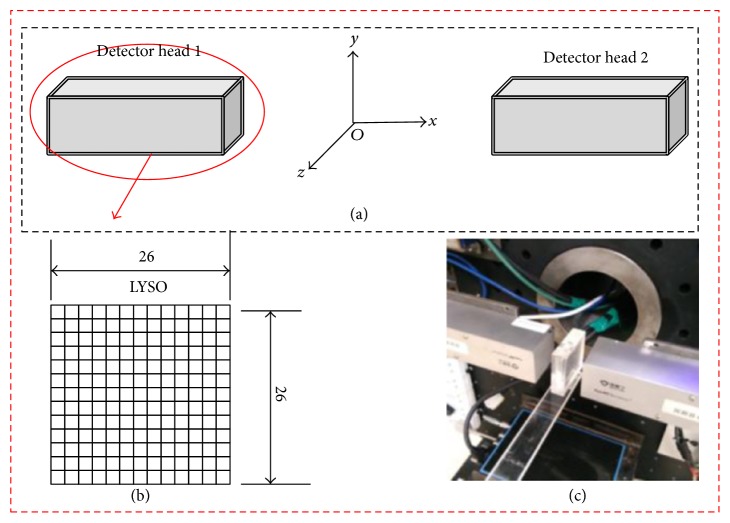
System structure of the dual-head PET system. (a) Geometry structure of the system and coordinate system. (b) Crystals of the system. (c) Prototype system.

We used the software package of GATE 6.2 [21, 22] to simulate the characteristics of a dual-head PET system. Two pixelated planar detector heads were designed in opposing positions. Each detector head contained 26 × 26 LYSO crystals with the size of 1.89 × 1.89 × 13 mm^3^. The pitch of each crystal was 2.038 mm. The system geometry and coordinate system are shown in Figures 1(a)-1(b). The *x*-axis is vertical to the detector heads and the *y*-axis is vertical to the ground. The other direction is defined as the *z*-axis, which is also the rotation axis. In this study, the system parameters were based on the prototype system built in our lab, as shown in Figure 1(c).

The back-to-back sources, where the two annihilation photons were generated at 180°, were used in the simulation. All sources were monoenergetic and no radioactive decays were considered. The energy resolution was set to 15% at 511 keV. The energy window was set from 350 to 650 keV and the timing coincidence window was 10 ns.

#### 2.1.2. Rotation Description

**Figure 2:**
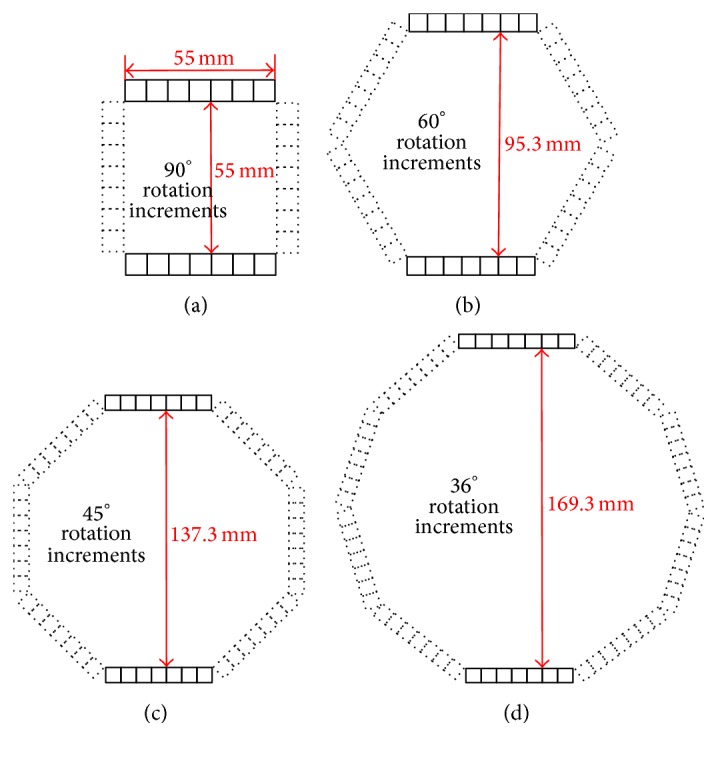
Distances between two nonoverlapping detector heads in space.

In the simulation, the rotation was carried out in a step-and-shoot mode and four different rotation increments were considered. The rotation increments (90°, 60°, 45°, and 36°) and the corresponding scanning positions were listed in Table 1. The separation distance between the two heads also could affect the imaging performance of the system. In order to compare the effects of the separation distance, we set the distance at 50 mm, 70 mm, and 100 mm, respectively. Furthermore, we calculated the distances which would keep the detector heads from overlapping in space. The calculated results were shown in Figure 2.

#### 2.1.3. Phantom Description

**Figure 3:**
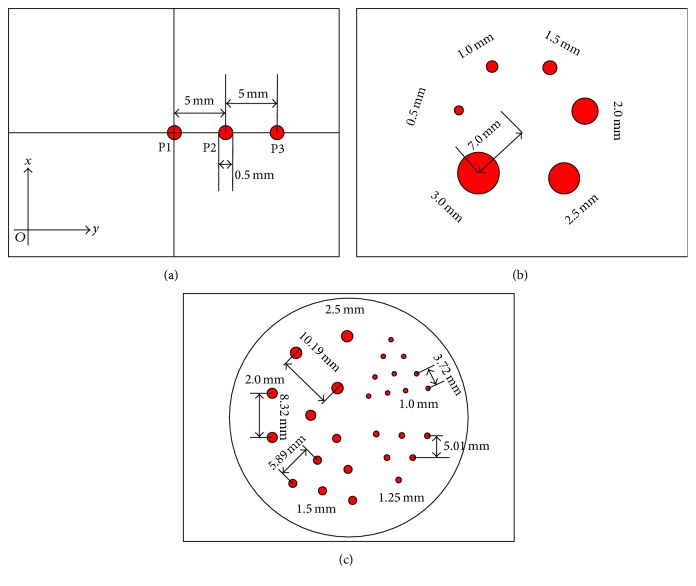
Simulation phantoms. (a) Three-point sources phantom. (b) Hot spot phantom. (c) Derenzo phantom.

Three types of phantoms (point phantom, hot spot phantom, and Derenzo phantom) were utilized to assess the effect of different rotation increments, and their structures were shown in Figure 3. The total coincidence events of different distances for these phantoms were listed in Table 2.

*Point Phantom*. Three-point sources with a diameter of 0.5 mm, which were denoted as P1, P2, and P3 in Figure 3(a), were simulated to characterize the spatial resolution. These sources were placed at 0 mm, 5 mm, and 10 mm away from the center of the (FOV) in the *y*-direction, respectively.

*Hot Spot Phantom*. As shown in Figure 3(b), six hot spots with a diameter of 0.5 mm, 1.0 mm, 1.5 mm, 2 mm, 2.5 mm, and 3.0 mm were inserted into the cold background phantom. The phantom was 0.5 mm in height and all the spots were 7 mm away from the FOV center.

*Derenzo Phantom*. The Derenzo phantom included in GATE was used in our simulation. It contained 5 groups of hot rods with a diameter of 1.0 mm, 1.2 mm, 1.5 mm, 2.0 mm, and 2.5 mm, respectively, as shown in Figure 3(c). The overall diameter of the phantom was 30 mm and 0.5 mm in height. During the simulation, the phantom was placed in the center of the FOV and its axis was parallel to the detector heads.

### 2.2. Reconstruction for the Rotatory Dual-Head PET System

In order to describe the transmission of the *γ* rays accurately, we calculated the system response matrix by MC simulations with GATE. More specifically, the voxel-based symmetry properties of the dual-head PET configuration were explored to reduce the simulation time [23]. The readers could consult our previous work [7] for details.

**Figure 4:**
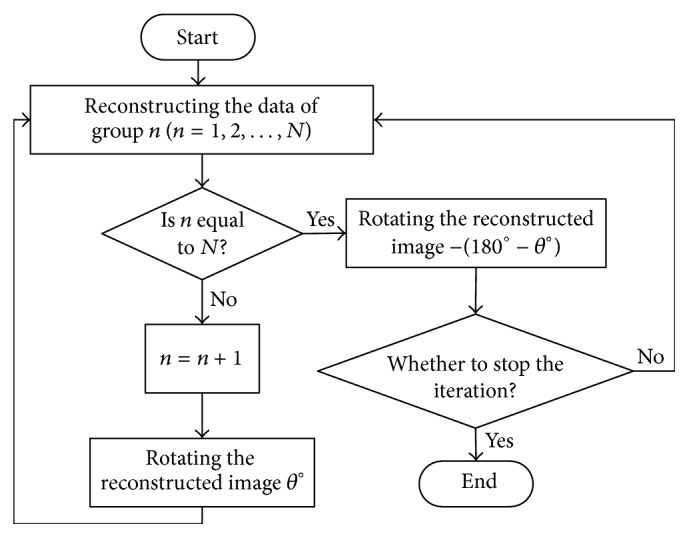
Main reconstruction flowchart.

The precalculated SRM fixed the relationships between the voxels and the LORs. When the system was rotated by an angle, the reconstructed images were in need of rotating by the same angle to match the precalculated SRM. In order to fuse the multiangle data together, we proposed a reconstruction scheme as shown in Figure 4. We supposed that the rotation increment was *θ* and the scanning position number was *N*, where *Nθ* = 180. Thus, we divided the scanning data into *N* groups based on the scanning position. For each group, the ordered subset expectation maximization (OSEM) [24] with five subsets was applied to reconstruction. After the reconstruction of one group was finished, we rotated the slices perpendicular to the rotation axis *θ*° and then the rotated results acted as the initial matrix of reconstruction for the next group data. At the end of each iteration, the rotated angles were adjusted to −(180° − *θ*°) to match with the reconstructed images of the first group data. Then, the iterative reconstruction would be stopped if the end condition was met; otherwise the *N* group data were reconstructed again.

When an image was rotated some angles which were not a multiple of 90°, an interpolation method was needed. In this study, we applied the nearest interpolation, bilinear interpolation, and bicubic interpolation.

### 2.3. Image Quality Assessment

Five metrics, including spatial resolution, normalized mean squared error (NMSE), peak signal-to-noise ratio (PSNR), contrast-to-noise ratio (CNR), and structure similarity (SSIM), were applied to assess the quality of the reconstructed images.

The SSIM index measured the similarity between two images [25, 26]. The spatial resolution was characterized by the full width at half maximum (FWHM) [27] of the point sources. The NMSE [28] was defined as follows:

(1) $$\begin{array}{c} \text{NMSE}=\frac{\sum_{m=0}^{M-1}\sum_{n=0}^{N-1}{\left(f\left(m,n\right)-t\left(m,n\right)\right)}^{2}}{\sum_{m=0}^{M-1}\sum_{n=0}^{N-1}t\left(m,n\right)}, \end{array}$$

where *f*(*m*, *n*) and *t*(*m*, *n*) were the pixel values of the reconstructed image and true image, respectively. *M* and *N* were the number of rows and columns in the images.

The PSNR [29] was calculated as follows:

(2) PSNR=10 log10⁡MAX21/MN∑m=0M−1∑n=0N−1fm,n−tm,n2.

Herein, MAX was the maximum possible pixel value.

In (3), the CNR [30, 31] was defined. *μ*_roi_ and *μ*_back_ denoted the mean value of the ROI and background in the reconstructed image, respectively; *δ*_roi_ and *δ*_back_ denoted their standard deviations.

(3) $$\begin{array}{c} \text{CNR}=\frac{\left|\mu_{\text{roi}}-\mu_{\text{back}}\right|}{\sqrt{\left(\delta_{\text{roi}}^{2}+\delta_{\text{back}}^{2}\right)/2}}. \end{array}$$

## 3. Results

For all experiments, the size of the reconstructed image matrix was 64 × 64 × 64 with a cubic voxel of 0.5 mm × 0.5 mm × 0.5 mm. Therefore, the entire reconstructed FOV was 32 × 32 × 32 mm^3^.

### 3.1. Accuracy of Different Interpolation Methods

In this subsection, we compared the performance of the different interpolation methods based on the point phantom and hot phantom. The reconstructed point phantom image provided the spatial resolution information, and the hot phantom was used to calculate the SSIM. For simplicity, we only used data simulated in the condition of 45° rotation increments with 100 mm distance between the two detectors.

**Figure 5:**
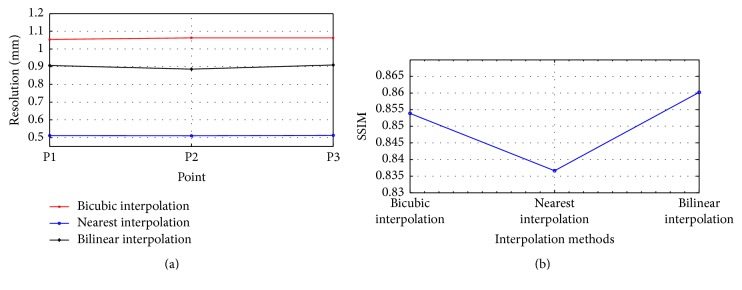
Resolution and SSIM with 45 rotation increments for different interpolation methods. (a) The results of resolution. (b) The results of SSIM.

As shown in Figure 5(a), the nearest interpolation method achieves the higher spatial resolution than the other two interpolation methods. In Figure 5(b), the bilinear interpolation method yields the highest SSIMs. Compared with the other interpolation methods, the bicubic interpolation performed poorly in both resolution and SSIM. Therefore, we only used and compared the nearest and bilinear interpolation in Sections 3.2 and 3.3.

### 3.2. Resolving Capability

The system's resolving capability was verified based on two phantoms: point phantom and Derenzo phantom. The point phantom was utilized to afford the quantitative evaluations, and the Derenzo phantom was mainly used to provide the qualitative assessments. In the quantitative analysis, five rotation increments were considered with three distances (50 mm, 70 mm, and 100 mm), and only the nearest interpolation method was used. In the qualitative analysis, the reconstructed results of 90° and 60° rotation increments were displayed. Furthermore, the results using the nearest and bilinear interpolation were compared in the condition of 60° rotation increments.

#### 3.2.1. Resolution Based on the Point Phantom

As the rotation operation mainly made the spatial resolution perpendicular to the detector heads (*x*-direction) improve greatly, we only calculated and compared the spatial resolutions along *x*-direction. The spatial resolutions of points P1, P2, and P3 are listed in Table 3. The nonrotated results (0°) were regarded as the reference to illustrate the advantages of the rotation operation. The results show that using the 60° rotation increments achieves the best spatial resolution. The resolutions increase about 0.15 mm~0.25 mm compared with that of the 90° and 45° rotation increments. In addition, the resolution of 36° and 45° rotation increments are almost similar to each other. Therefore, the 36° rotation increments would not be considered in the following sections to simplify the experiments.

#### 3.2.2. Reconstruction Results of the Derenzo Phantom

**Figure 6:**
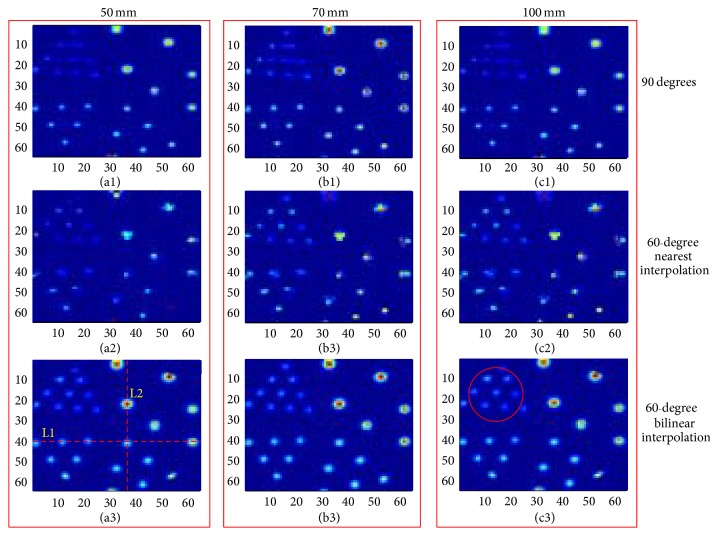
Reconstructed images of the Derenzo phantom. Images in the first, second, and third rows are reconstructed with 90° rotation increments and 60° rotation increments based on the nearest interpolation and bilinear interpolation, respectively. The columns, from the left to right, show the images from 50 mm, 70 mm, and 100 mm.

**Figure 7:**
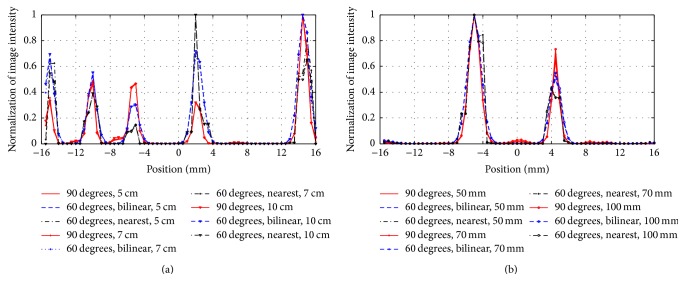
Profile curves along the red line in Figure 6(a1). (a) The profile along line L1. (b) The profile along line L2.

Figure 6 displays the reconstructed images of the Derenzo phantom. Images in the first, second, and third rows are reconstructed with 90° rotation increments and 60° rotation increments based on the nearest interpolation and bilinear interpolation, respectively. The columns, from the left to right, show the images from 50 mm, 70 mm, and 100 mm. We can find that the results of 60° rotation increments performed better than the results of 90° in terms of the resolving capability, especially in the region marked by the red circle. However, the results of 60° rotation increments with the nearest interpolation show an obvious image distortion, which is not shown in the bilinear interpolation method. Furthermore, we normalized the image intensity along the two red lines in Figure 6(c1) and drew the corresponding profiles in Figure 7. The profiles further illustrated that the nearest interpolation was not a suitable choice for the rotatory PET system.

### 3.3. Noise Level

In Section 3.2, the system's resolving capability was compared between different rotation increments. In this subsection, we further discussed the noise level for different rotation increments in terms of NMSE, PSNR, CNR, and SSIM. The hot spot phantom with three rotation increments (90°, 60°, and 45°) was considered for three distances (50 mm, 70 mm, and 100 mm). The noise levels of the reconstructed images using the nearest and bilinear interpolation were evaluated.

**Figure 8:**
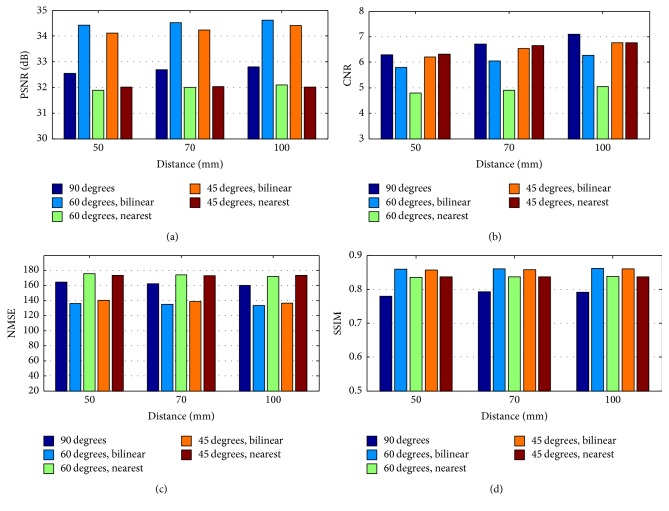
Results of different rotation increments with bilinear and nearest interpolations. (a) PSNR. (b) CNR. (c) NMSE. (d) SSIM.

The results are shown in Figure 8. From the view of the interpolation method, the nearest interpolation method has no advantage over the bilinear interpolation in all of the metrics. In view of the rotation increments, the results of the 45° rotation increments are slightly worse than those of the 60° rotation increments except for the CNR, but the difference is not obvious. The results of the 90° rotation increments are more advantageous in CNR than for the 60° rotation increments, but they have no advantages in terms of PSNR, NMSE, and SSIM.

**Figure 9:**
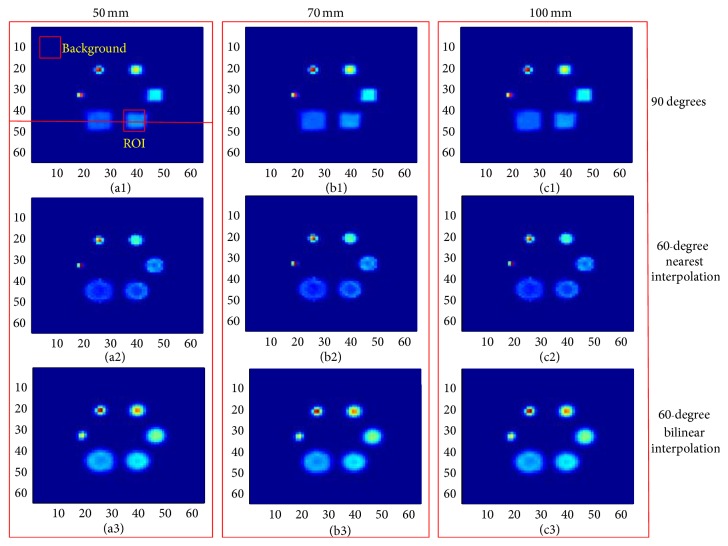
Reconstructed images of the hot spot phantom. The first row is the reconstructed results of 90° increments. The second and the third row are the results of 60° increments based on the nearest interpolation and bilinear interpolation, respectively. The columns, from the left to right, show the images from 50 mm, 70 mm, and 100 mm, respectively.

**Figure 10:**
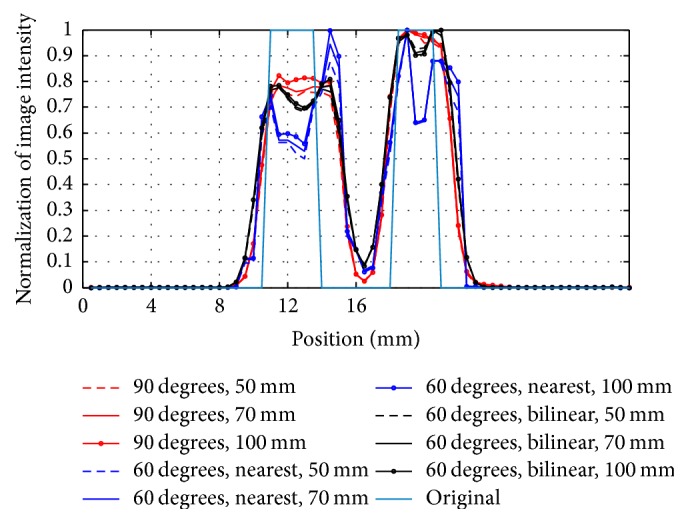
Profiles of normalization of image intensity along the red line in Figure 9.

Furthermore, the reconstructed images of the hot spot phantom are displayed in Figure 9. The first row is the reconstructed results of 90° rotation increments. The second and the third row are the results of 60° rotation increments based on the nearest interpolation and bilinear interpolation, respectively. The columns, from the left to right, show the images from 50 mm, 70 mm, and 100 mm. The profile curves along the red line in Figure 9(a1) are shown in Figure 10. From the profile curves, it can be observed that the profile curves of 90° rotation increments are uniform compared to that of 60° rotation increments.

Overall, the 60° and 90° rotation increments had advantages over the other increments. The reconstructed images of 60° rotation increments had advantages in PSNR, NMSE, and SSIM. In terms of CNR, the reconstructed images of 90° rotation increments could achieve better results. We also found that the reconstructed images with 90° rotation increments were uniform by observing. Therefore, both 90° and 60° rotation increments could be used in the real experiments, and which one to choose might depend on the application requirement.

### 3.4. Real Experiment

**Figure 11:**
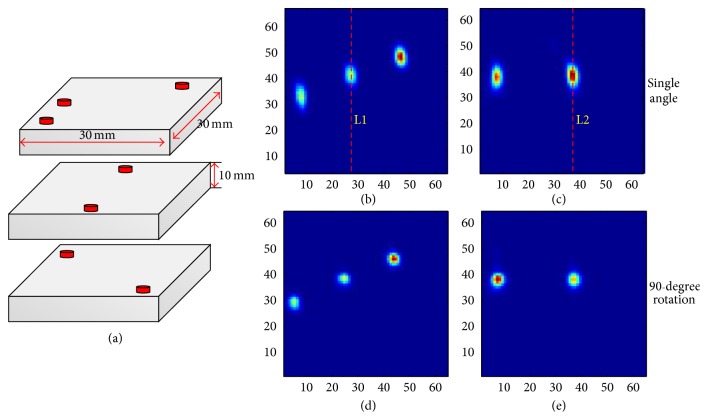
(a) Structure of the phantom. (b) and (c) are the reconstructed slices from single angle (nonrotated). (d) and (e) are the results with the 90° rotatory operation.

A phantom with 7 hot points with a diameter of 2 mm was designed as shown in Figure 11(a). It was made of polymethyl methacrylate (PMMA) and composed of three parts. Each part was 30 × 30 × 10 mm^3^ and contained 3, 2, and 2 hot points, respectively. In the experiment, 20 *μ*Ci ^18^F-FDG solution was poured into these hot points and then the three parts were stacked together. The phantom was placed in the center of the FOV. Considering the size of this phantom, the distance between the detector heads was set to 50 mm. In this situation, the 90° rotation increments would be enough to maintain the completeness of the forward data; thus, we utilized the 90° rotation in the experiment. The scanning time lasted 10 min for each position.

**Figure 12:**
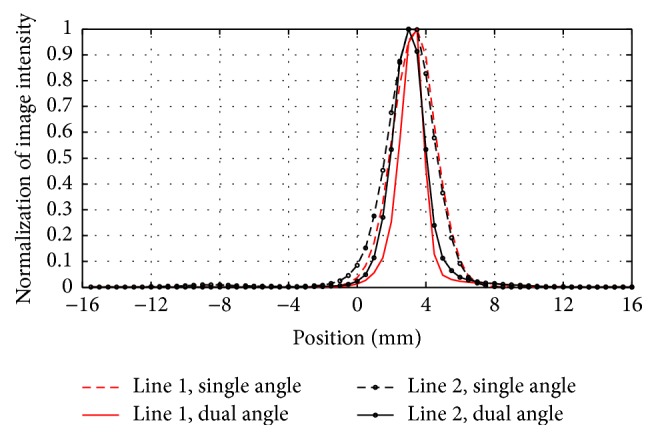
Profiles along the two red lines in Figure 11(b).

Figures 11(b)–11(e) show the reconstructed images of the phantom. Figures 11(b) and 11(c) are the reconstructed slices of forward data from single angle (nonrotation). Figures 11(d) and 11(e) are the results with the 90° rotatory operation. The results of a single angle (nonrotation) were taken as a reference.  Figure 12 shows the profile along the two red lines in Figures 11(b) and 11(c). By comparing and analyzing the reconstruction results, we could find that the 90° rotation operation could solve the imaging stretch problem effectively.

## 4. Discussion and Conclusion

In this study, we evaluated the influence of the different rotation increments on imaging performances of a rotatory dual-head PET system. Five rotation increments were compared in the simulation. In the real experiment, we further evaluated the imaging performance of the dual-head PET system with 90° rotation increments.

In order to fuse the forward data of different acquired positions, a reconstruction flowchart was proposed based on a precalculated SRM which was obtained by MC simulation. For the precalculated SRM, the relationships between the voxels and LORs were fixed. Therefore, we added the image interpolation methods to the reconstruction flowchart. The bicubic interpolation performed worse in both resolution and SSIM as shown in Section 3.1. That may be caused by lots of negative pixels emerging in the images after bicubic interpolation. Those negative pixels are not matched with the OSEM iteration algorithm which has a nonnegativity constraint.

The resolution was evaluated to verify the system's resolving capability without and with rotation operation in Section 3.2. The quantitative and qualitative assessments were evaluated based on the point phantom and the Derenzo phantom, respectively. For the dual-head PET system, the resolutions perpendicular to the detector heads are very low, which can illustrate the effect of the rotation operation. Therefore, only the resolutions in this direction were calculated. By comparing and analyzing the resolution and the reconstructed images, we find that the rotation operation is effective for the dual-head PET system.

The NMSE, PSNR, CNR, and SSIM were applied to evaluate the noise level of the reconstructed images with different rotation increments. In this aspect, the 90° and 60° rotation increments had advantages over the other increments. In terms of the NMSE, PSNR, and SSIM, the 60° rotation increments could obtain the best results. In terms of CNR, the 90° rotation increments were better than other increments. In addition, the reconstructed images with 90° rotation increments were uniform as shown in Figures 9 and 10. Therefore, it is reasonable to take the merits as a reference, and a desired application should also be taken into consideration when determining the rotation increments.

In the simulation experiments, we did not consider the effects of radioactive decays, system dead-time and scatter, or such other physical factors. We also did not consider the effect of mechanical installation. However, those factors cannot be ignored in practice. Therefore, the 90° rotation operation was conducted in the real experiments. In our future work, we will provide the system performance of a prototype dual-head PET system with 90° rotation in more detail. A series of in vivo and in vitro experiments will be carried out based on the prototype system.

## Figures and Tables

**Table 1:** Rotation increments and corresponding scanning positions.

Rotation increments	Scanning positions
0°	0°
90°	0° → 90°
60°	0° → 60° → 120°
45°	0° → 45° → 90° → 135°
36°	0° → 36° → 72° → 108° → 144°

**Table 2:** Total coincidence events of different distances for phantoms.

	50 mm	70 mm	100 mm
Point phantom	3.4 × 10^5^	2.2 × 10^5^	1.3 × 10^5^
Hot spot phantom	2.4 × 10^7^	1.4 × 10^7^	8.9 × 10^6^
Derenzo phantom	1.0 × 10^7^	6.8 × 10^6^	4.2 × 10^6^

**Table 3:** Resolutions with different rotation increments with the nearest interpolation.

	0°	90°	60°	45°	36°
100 mm					
P1	3.93	0.68	0.51	0.65	0.61
P2	3.95	0.71	0.62	0.66	0.62
P3	4.01	0.69	0.51	0.66	0.63

70 mm					
P1	3.16	0.71	0.48	0.59	0.55
P2	3.15	0.71	0.49	0.61	0.56
P3	3.18	0.72	0.48	0.60	0.56

50 mm					
P1	3.04	0.79	0.52	0.69	0.65
P2	3.04	0.78	0.53	0.69	0.65
P3	3.08	0.80	0.54	0.71	0.66
